# Single-cell analysis of white adipose tissue reveals the tumor-promoting adipocyte subtypes

**DOI:** 10.1186/s12967-023-04256-7

**Published:** 2023-07-15

**Authors:** Si-Qing Liu, Ding-Yuan Chen, Bei Li, Zhi-Jie Gao, Hong-Fang Feng, Xin Yu, Zhou Liu, Yuan Wang, Wen-Ge Li, Si Sun, Sheng-Rong Sun, Qi Wu

**Affiliations:** 1 Department of Breast and Thyroid Surgery, Renmin Hospital of Wuhan University, Wuhan, Hubei People’s Republic of China; 2 Department of Pathology, Renmin Hospital of Wuhan University, Wuhan, Hubei People’s Republic of China; 3 Department of Breast and Thyroid Surgery, Huangshi Central Hospital, Hubei Polytechnic University, Huangshi, Hubei People’s Republic of China; 4 Department of Oncology, Shanghai Artemed Hospital, Shanghai, People’s Republic of China; 5 Department of Clinical Laboratory, Renmin Hospital of Wuhan University, Wuhan, Hubei People’s Republic of China; 6 Tongji University Cancer Center, Shanghai Tenth People’s Hospital, Tongji University School of Medicine, Shanghai, People’s Republic of China

**Keywords:** Tumor-adipose microenvironment, Single-cell RNA sequencing, Cancer-associated adipocytes, Adiponectin receptor

## Abstract

**Background:**

The tumor-adipose microenvironment (TAME) is characterized by the enrichment of adipocytes, and is considered a special ecosystem that supports cancer progression. However, the heterogeneity and diversity of adipocytes in TAME remains poorly understood.

**Methods:**

We conducted a single-cell RNA sequencing analysis of adipocytes in mouse and human white adipose tissue (WAT). We analyzed several adipocyte subtypes to evaluate their relationship and potential as prognostic factors for overall survival (OS). The potential drugs are screened by using bioinformatics methods. The tumor-promoting effects of a typical adipocyte subtype in breast cancer are validated by performing in vitro functional assays and immunohistochemistry (IHC) in clinical samples.

**Results:**

We profiled a comprehensive single-cell atlas of adipocyte in mouse and human WAT and described their characteristics, origins, development, functions and interactions with immune cells. Several cancer-associated adipocyte subtypes, namely DPP4^+^ adipocytes in visceral adipose and ADIPOQ^+^ adipocytes in subcutaneous adipose, are identified. We found that high levels of these subtypes are associated with unfavorable outcomes in four typical adipose-associated cancers. Some potential drugs including Trametinib, Selumetinib and Ulixertinib are discovered. Emphatically, knockdown of adiponectin receptor 1 (AdipoR1) and AdipoR2 impaired the proliferation and invasion of breast cancer cells. Patients with AdipoR2-high breast cancer display significantly shorter relapse-free survival (RFS) than those with AdipoR2-low breast cancer.

**Conclusion:**

Our results provide a novel understanding of TAME at the single-cell level. Based on our findings, several adipocyte subtypes have negative impact on prognosis. These cancer-associated adipocytes may serve as key prognostic predictor and potential targets for treatment in the future.

**Supplementary Information:**

The online version contains supplementary material available at 10.1186/s12967-023-04256-7.

## Background

Adipose tissue is a highly metabolic organ that regulates energy balance in the body. In the context of cancer, adipose tissue can be found in close proximity to tumors and tumors can grow in the vicinity of or metastasize to adipose tissue, which helps to form a unique tumor microenvironment called TAME. TAME link tumor growth with obesity and other metabolic disorders [1]. Different types of non-malignant cells are present in the TAME, mainly containing endothelial cells, adipocytes, immune cells, and other stromal cells. These stromal cells interact with malignant cells and contribute to tumor progression, metastasis, angiogenesis and therapeutic resistance. Mechanistically, the complex and dynamic pathways like deposition of extracellular matrix, metabolic regulators, cytokine responses and immunological states are responsible for tumor development.

WAT is the type of fatty tissue found at anatomic sites. The major WAT depots are classified according to their anatomic location as either subcutaneous adipose tissue (SAT) or visceral adipose tissue (VAT). Adipocytes, as the most dominant component of WAT, are consist of mature adipocytes and adipose stem and progenitor cells (ASPCs), which are the cells that give birth to mature adipocytes [2]. Similarly, adipocytes are the predominant component in the TAME, in which tumor cells co-opt adipocytes, converting them into cancer-associated adipocytes (CAAs) [1]. CAAs have been proved to play tumor-promoting role in diverse type of cancers [3–5]. CAAs create a tumor-favoring ecosystem via supplying high-energy metabolites, recruiting immunosuppressive cells, impairing the functions of T cells and activating angiogenesis with endothelial cells [6]. Hence, CAAs display the potential value as a prognostic factor and therapeutic target. For example, in the case of pancreatic cancer, the transformation of adipocytes into CAAs has been demonstrated to enhance malignant traits through the expression of SAA1[4]. Likewise, in breast cancer, CAAs exert a tumor-promoting effect by releasing multiple adipokines such as leptin and adiponectin [3, 6]. However, the heterogeneity and plasticity of CAAs in type-specific cancer remain unclear. Likewise, the definitive origin of CAAs and potential crosstalk between CAAs and other TAME components during cancer progression requires further investigation.

The advent of single-cell RNA sequencing (scRNA-seq) has provided unprecedented opportunities to identify and characterize the components of WAT based on mouse and human models. Since the mature adipocytes are incompatible with traditional single-cell approaches. An alternative strategy named single-nucleus RNA sequencing (snRNA-seq), which can capture mature adipocytes, has also been used to describe WAT [7, 8]. Meanwhile, increasing scRNA-seq strategies have been developed to profile the characteristics of cancer cells and their micro-ecosystem. However, the potential composition, interrelationship, and generalized characterization of CAA at single-cell resolution remain lacking.

To address these outstanding questions, we conducted a comprehensive single-cell atlas of adipocytes in mouse and human WAT. We systematically mapped the adipocyte subpopulations and subsequently revealed molecular profiles of several type-specific CAAs. The potential drugs are further screened. Finally, we depicted the tumor-promoting effects of ADIPOQ^+^CAAs on malignant behaviors of breast cancer by performing functional assays in vitro and prognostic analysis in clinical samples. In conclusion, our systematic investigation of CAAs and their subtypes across cancers at single-cell resolution highlights the possible plasticity and heterogeneity of CAAs in cancer biology and proposes a future treatment target.

## Methods

### **scRNA-seq and snRNA-seq datasets**

The snRNA-seq datasets of mouse visceral adipose tissue cells were acquired from the Gene Expression Omnibus (GEO) database (GSE160729 and GSE176171) [8, 9]. The scRNA-seq datasets of human visceral adipose tissue cells were acquired from the GEO database (GSE189783, GSE136229 and GSE129363) [10]. The snRNA-seq datasets of mouse subcutaneous adipose tissue cells were acquired from the GEO database (GSE180589, GSE133486 and GSE176171) and ArrayExpress database (E-MTAB-6677) [8, 11–13]. The scRNA-seq datasets of human subcutaneous adipose tissue cells were acquired from the GEO database (GSE155960, GSE128890, GSE129363 and GSE176067) [8, 10, 14, 15].

### scRNA-seq and snRNA-seq data processing

The scRNA-seq and snRNA-seq data were processed for quality control, dimension reduction and unsupervised clustering by following the workflow in Seurat [16]. Each sample was individually quality checked, and cells were filtered to ensure good gene coverage, a consistent range of read counts and low numbers of mitochondrial reads. At least 200 and no more than 6000 detected gene were required for each cell. No more than 15% mitochondrial reads were allowed per cell. Due to the multiple sources of the data, they used different cell dissociation and handling protocols, library-preparation technologies or sequencing platforms. All of these factors result in batch effects, in which the expression of genes in one batch differs systematically from that in another batch. Thus, to integrate data from different datasets, we performed per-cell size-factor normalization for each sample while per-gene z-score scaling across cells were not performed. Subsequently, a batch effect correction algorithm, fastMNN, was applied to correct the batch effect among datasets. The fastMNN was conducted by using the RunFastMNN function from the SeuratWrappers package of R with default parameters [17]. On the basis of the fastMNN result, downstream analyses including dimensionality reduction and clustering were conducted using the Seurat package of R.

### Pathway enrichment

Seurat function FindAllMarkers with the Wilcox test was used to identify differentially expressed genes (DEGs) for each cell subpopulation. Genes with p value < 0.05 and log2 fold change > 0.5 were considered as DEGs. DEGs were used as input into the ClusterProfiler package of R to conduct KEGG (Kyoto Encyclopedia of Genes and Genomes) pathway enrichment analyses. The visualization of the results was conducted by ggplot2 package of R.

### Pseudo-time analysis

To determine the dramatic translational relationships among each subpopulation, we applied the pseudo-time analysis with Monocle2 using DDR-Tree and default parameters [18]. Positive marker genes for each subpopulation were used. Based on the pseudo-time analysis, branch expression analysis modelling was applied for branch fate determined gene analysis.

### Transcription factor analysis

The Dorothea resource was used to infer transcription factor (TF) activity [19]. We chose ‘A’, ‘B’ and ‘C’ high-confidence TF selection. Viper scores were computed on the regulons. We computed the mean and standard deviation values of the scaled viper scores per severity group for the comparison of TF score activities. TFs were ranked according to the variance of their corresponding viper scores. The highly variable scores per severity group (n = 150 TFs in total) were kept for visualization of their corresponding scores.

### Cell–cell interaction analysis

Cell–cell interaction analyses was performed by using CellChat R package [20]. We followed the official workflow and default parameter settings to load the adipocyte and immune cell population into CellChat after quality inspection and normalization. The built-in CellChatDB.mouse database was used as a reference for screening receptor-ligand interactions. The number and strength of potential ligand-receptor interactions between cells were calculated using computeCommunProb, computeCommunProbPathway and aggregateNet functions with standard parameters. The interaction scores were estimated between cell types. The number of ligand-receptor pairs between every two cell types was shown in the form of heatmap. The weights/strength of ligand-receptor pairs between every two cell types was shown in the form of circos plot.

### Deconvolutions of bulk RNA-seq transcriptomics

DWLS (Dampened weighted least squares) estimation method was used to deconvolute predicted cell fractions from a number of bulk transcript profiling datasets [21]. The Cancer Genome Atlas (TCGA) expression matrices and clinical information were obtained from UCSC Xena [22]. The TCGA samples were grouped by predicted cell scores. Once the estimated proportion of a subtype is larger than 0.5, that sample will be placed into the group of representing the subtype. Group mixture contained samples with multiple subtype components.

### Survival analyses

Differences in survival among groups were assessed using Kaplan–Meier analysis and log-rank test statistics using the survival and survminer R packages. In addition, patients in TCGA cohort were separated into low-expression and high-expression groups based on the best cutoff calculated by the survminer package of R. Univariate and multivariate Cox regression analysis was performed in TCGA breast cancer cohort using the coxph function in survival package of R. The forest plots were conducted by the ggforest function in ggplot2 package of R.

### Drug sensitivity prediction

Drug response of each TCGA breast cancer sample was estimated based on GDSC2 (Genomics of Drug Sensitivity in Cancer) and CTRP2 (Cancer Therapeutics Response Portal) database by using the R package OncoPredict [23]. Pearson’s correlation of inhibitor response (measured by IC50) with each of the four adipocyte subtype scores was calculated. All drugs displayed in the heatmap showed a significant correlation with at least one subtype.

### Cell culture and reagents

The human breast cancer cell lines ZR751, MCF-7 and MDAMB-231 cells were obtained from American Type Culture Collection (ATCC, Shanghai). The human breast cancer cell lines ZR751, MCF-7 and MDAMB-231 were cultured in Dulbecco’s modified Eagle’s medium (DMEM) supplemented with 10% FBS and 1% penicillin–streptomycin in a humidified 37 °C incubator with 5% CO2.

### Western blot assay

Cells or tissues are lysed in RIPA lysis buffer containing a mixture of intact protease and phosphatase inhibitors. A total 30 µg of protein per grouped sample was loaded onto SDS-PAGE gels and subsequently transferred onto PVDF membranes. Then the membranes were loaded and incubated with primary antibodies (AdipoR1, AdipoR2 and tubulin) at 4 °C for overnight. The membrane was then incubated with horseradish peroxidase-conjugated secondary antibody for 2 h at room temperature. At last, Images were captured with ChemiDocTM Imaging System. The primary and secondary antibodies used for WB were from Thermo Fisher Scientific.

### Transwell assay

The migration and invasion ability of MDA-MB-231 cells were analyzed using a 24-well transwell chamber with polycarbonate membranes and matrigel. Cells were inoculated in the upper chambers with serum-free DMEM, and the lower chamber was filled with 10% FBS. Cells that passed through the membranes were fixed with paraformaldehyde and dyed with Crystal Violet after incubating for 48 h. The cells in the lower chambers were photographed and counted.

### Flow cytometry

The fraction of cells undergoing cell death in response to shRNA lentivirus was quantified by flow cytometry using an Annexin V-FITC/PI Apoptosis Kit (Elabscience Biotechnology). All procedures were conducted according to the manufacturer’s instructions. Last, the data was recorded by Beckman Coulter CytoFLEX and analyzed using the CytExpert software.

### Relative growth assay

Relative growth rates were estimated by the MTT assay. Approximately 5 × 103 cells were seeded in 96-well plates with 100 µl medium each well.

### Patients

We collected 83 pre-operative fasting blood sample from patients who were prepared for surgical treatment of breast disease at Renmin Hospital of Wuhan University between November 2016 and October 2017. The serum levels of adiponectin were measured using an enzyme-linked immunosorbent assay. We collected 113 patients who underwent surgical treatment at Renmin Hospital of Wuhan University between January 2010 and January 2014 and were diagnosed with invasive breast cancer by conventional pathology. This study was approved by the Ethics Committee of Renmin Hospital of Wuhan University. Written informed consent was obtained from all patients.

### IHC assay

The tissue samples were fixed, paraffin-embedded, dewaxed, rehydrated, and antigen retrieval. Then samples were stained with AdipoR1 and AdipoR2 antibody at 4 °C overnight, followed by incubation in secondary biotinylated antibody for 30 min at 37 °C, and finally visualized with DAB solution and counterstained with hematoxylin. Finally, photographs were taken under an optical microscope. Five representative images at 40× magnification was acquired for quantitative analysis by using ImageJ software.

### Statistical analysis

Statistical analyses were applied using GraphPad Prism (version 8.0). All experiments were performed at least three times independently. The results are presented as means ± SD. We used t test to compare data from two groups. Multiple comparisons between groups were performed using the Tukey’s multiple comparison test or Mann–Whitney U test. We used the Kaplan–Meier method to estimate survival probabilities for RFS and OS, and variables were compared using the log-rank test. In the quantitative analysis graphs, a single asterisk (*) indicated p < 0.05, two asterisks (**) indicated p < 0.01, and three asterisks (***) indicated p < 0.001.

## Results

### **A single-cell atlas of mouse and human WAT**

**Fig. 1:**
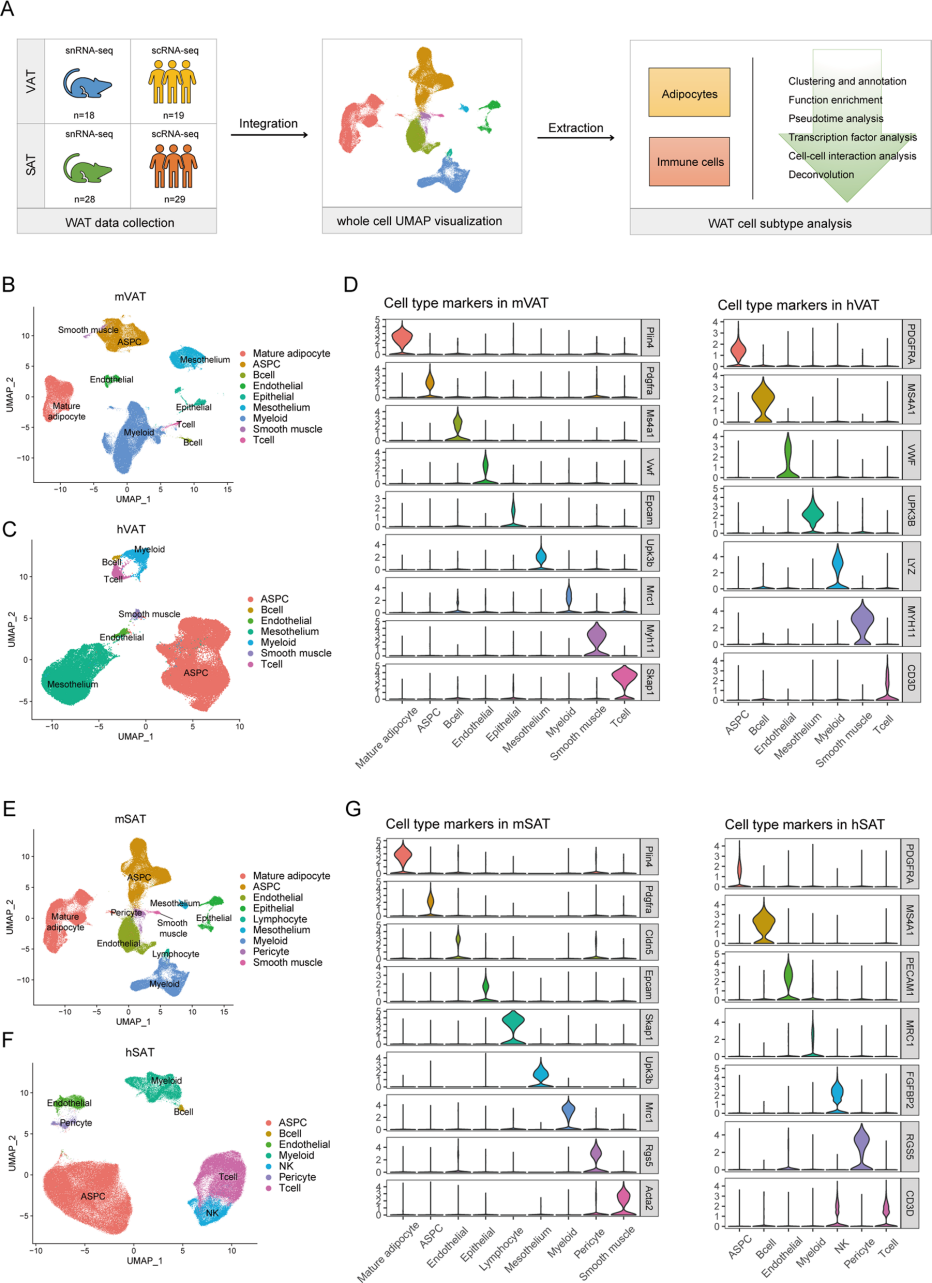
A single-cell atlas of mouse and human WAT. **A** Schematic of workflows for the scRNA-seq/snRNA-seq analysis of mouse and human WAT. **B** UMAP visualization of 121,349 nuclei from mouse VAT showing nine major cell types. **C** UMAP visualization of 53,152 cells from human VAT showing seven major cell types. **D** Violin plots of marker genes for each cell population in the mouse and human VAT datasets. **E** UMAP visualization of 158,977 nuclei from mouse SAT showing nine major cell types. **F** UMAP visualization of 144,965 cells from human SAT showing seven major cell types. **G** Violin plots of marker genes for each cell population in the mouse and human SAT datasets

To comprehensively understand the cellular composition of the TAME, we compiled a single-cell transcriptional atlas of mouse and human WAT samples (Fig. 1A). Our atlas included scRNA-seq data from 48 human samples (nineteen VAT samples and twenty-nine SAT samples) and snRNA-seq data from 46 mouse samples (eighteen VAT samples and twenty-eight SAT samples) [7, 8, 10–14]. After rigorous quality filtering of each sample, we sought to integrate the datasets into four groups: mouse VAT (mVAT), human VAT (hVAT), mouse SAT (mSAT) and human SAT (hSAT). Next, we conducted MNN integrations across the datasets to reduce the batch effects [17]. The resulting quality-controlled VAT single-cell atlas included 121,349 single nuclei and 53,152 single cells that were clustered based on canonical lineage markers and visualized using uniform manifold approximation and projection (UMAP) plots. Clustering analysis and marker gene annotation revealed distinct clusters, including mature adipocytes, ASPCs, endothelial cells, epithelial cells, mesothelial cells, and immune cells (T cells, B cells and myeloid cells) (Fig. 1B–D). In the single-cell atlas of SAT, low-quality filtering left 158,977 single nuclei and 144,965 single cells. The initial clustering visualized by UMAP and marker gene annotation revealed major cell populations, including mature adipocytes, ASPCs, vascular cells (pericytes and endothelial cells), epithelial cells, mesothelial cells and immune cells (T cells, B cells and myeloid cells) (Fig. 1E–G). The distribution of cell types among the mVAT, hVAT, mSAT, and hSAT atlases did not exhibit significant differences. However, since mature adipocytes cannot be distinguished using scRNA-seq approaches, they were hardly found in the hVAT and hSAT atlases [8].

### Landscape of the adipocyte population in mouse and human WAT

Next, we conducted detailed analyses of adipocyte populations (including ASPCs and mature adipocytes) in both VAT and SAT. Previous studies in mice suggested that ASPCs can be classified into three robust populations [24]. The first population, adipose stem cells (ASCs), is the most stem-like population in nature and marked by the expression of stem-related genes, such as Pi16, Dpp4 and Adamts16 [15]. The second population, preadipocytes (PreAs), expresses several adipogenesis-related genes, such as Lpl and Plin2. Preadipocyte is in a cell state committed toward adipogenesis [25]. The third population, adipogenesis regulators (Aregs), is defined by the expression of F3 (encoding CD142) and is capable of inhibiting the adipogenic differentiation of other ASPCs [26]. Mature adipocytes are considered to be essentially uniform in function, although some recent studies do not share this belief.

#### *VAT contains distinct subpopulations of adipocytes*

**Fig. 2:**
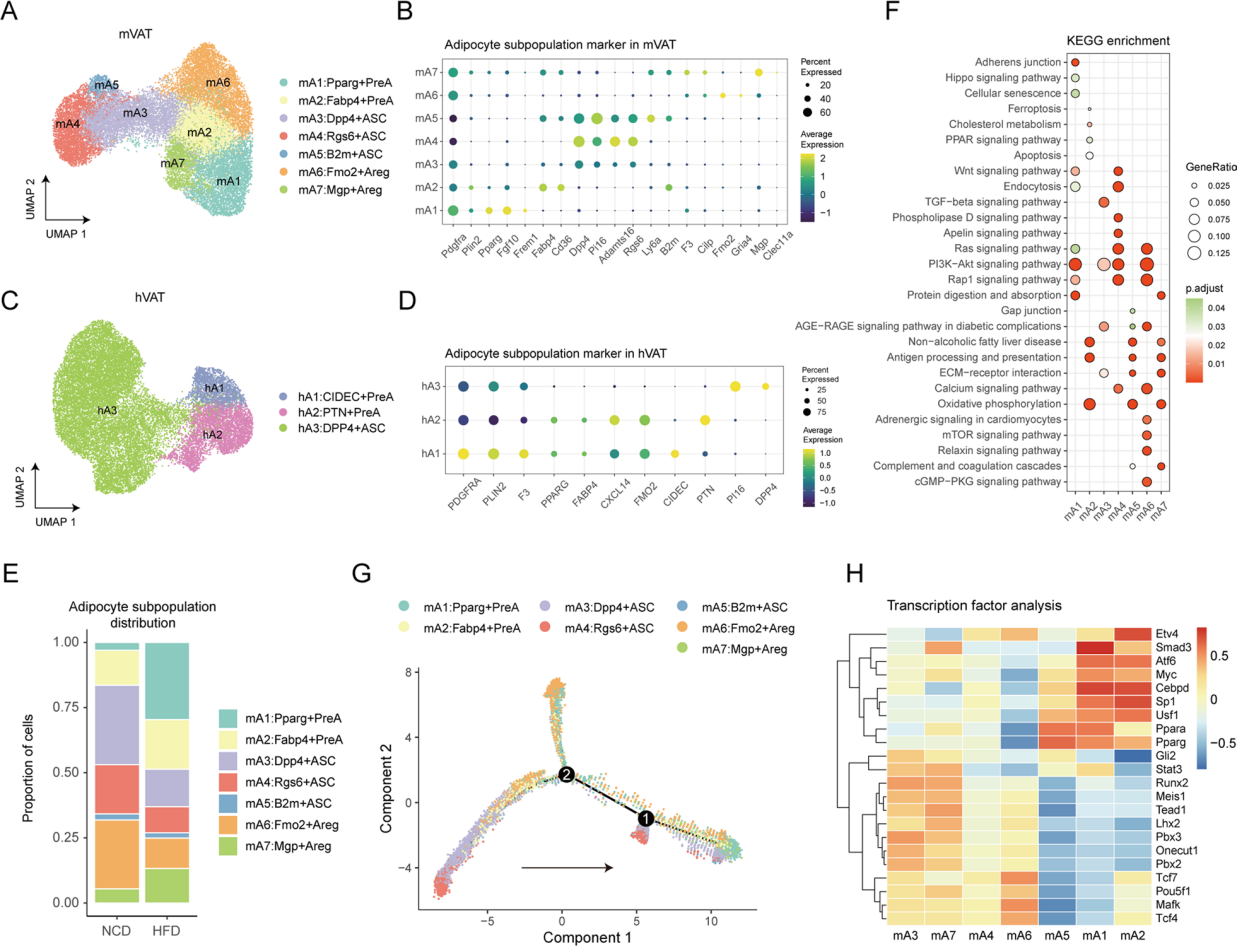
Landscape of the adipocyte population in mouse and human VAT. **A** UMAP visualization of inferred ASPCs from mouse VAT identified seven adipocyte subpopulations. **B** Dot plot of marker genes for each cell subpopulations in mouse ASPCs. **C** UMAP visualization of inferred adipocytes from human VAT identified three adipocyte subtypes. **D** Dot plot showing marker genes for each cell subpopulations in human adipocytes. **E** Relative proportions of cell subpopulations in mouse ASPCs from HFD mouse (n = 11) or NCD mouse (n = 8). **F** Dot plot showing the pathway enrichment of each cell subpopulation in mouse ASPCs using KEGG datasets. **G** Pseudo-time trajectory of cell subpopulations in mouse ASPCs. (H) Heatmap showing the highly variable TF activities among the cell subpopulations in mouse ASPCs.

In mVAT, we detected seven distinct subpopulations of ASPCs (marked by Pdgfra) and three subpopulations of mature adipocytes (marked by Plin4). Based on the previous conclusions and newly generated gene expression profiles, we annotated the seven ASPC subpopulations as mA1: Pparg^+^PreA (Pparg, Fgf10, Frem1), mA2: Fabp4^+^PreA (Fabp4, Cd36), mA3: Dpp4^+^ASC (Dpp4), mA4: Rgs6^+^ASC (Rgs6, Adamts16) mA5: B2m^+^ASC (Ly6a, B2m), mA6: Fmo2^+^Areg (Fmo2 and Gria4) and mA7: Mgp^+^Areg (Mgp and Clec11a) (Fig. 2A, B). We also identified three subpopulations of mature adipocytes as Cfd^+^, Lep^+^, and Prune2^+^ mature adipocytes by the upregulated expression of their marker genes (Additional file 1: Fig. S1A, B). Compared to mouse adipocytes, human adipocytes exhibit greater individual variability and less heterogeneity. Subclustering of human adipocytes revealed three populations, including hA1: CIDEC^+^PreA, hA2: PTN^+^PreA and hA3:DPP4^+^ASC (Fig. 2C, D). Aregs and mature adipocytes were not detected.

Obesity is known to profoundly impact the abundance and gene expression of adipocytes. Thus, we investigated the distribution of each subpopulation between high-fat diet (HFD) and normal control diet (NCD) mVAT samples (Fig. 2E, Additional file 1: Fig. S1C). The comparison analysis revealed that HFD-induced obesity resulted in an increase in the relative number of mA1, mA2 and mA7 cells, whereas the relative number of mA3 and mA6 cells was decreased (Fig. 2E). Notably, mature adipocytes were more influenced by diet than ASPCs were, as the relative proportions of Cfd^+^adipocytes were vastly reduced after HFD feeding, whereas the opposite trend was observed for Lep^+^adipocytes (Additional file 1: Fig. S1C). Notably, all adipocyte subpopulations were present in the majority of samples, suggesting that these subtypes are stable and do not reflect sample-specific variation.

A critical inquiry is whether adipocyte subpopulations have distinct functions. To address this question, we first explored the function of each subpopulation by performing pathway enrichment analysis (Fig. 2F). The three subpopulations of mature adipocytes have relatively few specific markers, and their features were found to be generic. We focused on subpopulations mA1–mA7 for a more detailed analysis. Both mA1 and mA2 expressed high levels of mature adipocyte marker genes, but they differed in function. The mA1 subpopulation was uniquely enriched for the Hippo signaling pathway, suggesting a dedifferentiated status of adipocytes. The Hippo pathway is known to mediate a shift of adipocytes from energy storage to extracellular matrix remodeling in adipose tissue fibrosis [27]. In comparison, mA2 adipocytes were characterized by enrichment of ferroptosis, apoptosis, cholesterol metabolism, and the PPAR signaling pathway. Among the ASC subpopulations, including mA3-5, mA3 was uniquely enriched in the TGF-beta signaling pathway, indicating an immune-related feature. M2-like macrophages in adipose tissue inhibit adipocyte progenitor proliferation via the TGF-beta signaling pathway [28]. Furthermore, mA4 was enriched in the phospholipase D and apelin signaling pathways, while mA5 features enrichment for gap junction. Compared with mA7, mA6 was more distinctly enriched in the mTOR signaling pathway, the relaxin signaling pathway and the CGMP-PKG signaling pathway (Fig. 2F). To further examine the potential trajectories of adipocyte subpopulations, we found that mA4 is the precursor of adipogenic commitment, as mA4 gradually differentiates into mA3, which in turn gives rise to mA1, mA2, and mA6. Interestingly, adipocytes from HFD-induced obese mice were fully distributed in the right arm, while others were situated in the left arm (Additional file 1: Fig. S1A). Since only a small proportion of the adipocytes in the right arm were derived from the left arm, we hypothesized that obesity directly influence origin subpopulations, including mA3 and mA4. Finally, we asked whether TFs contribute to the phenotypic state of these subpopulations (Fig. 2H). It has been reported that transcriptional diversity decreases during differentiation [29]. Our results confirmed that mA3 and mA4, rather than mA5, showed increased transcriptional diversity, consistent with their earlier differentiation state. However, the Areg subpopulation mA7 also showed increased transcriptional diversity, which suggested a dedifferentiation potential [30]. In addition, Smad3, an important contributor to the maintenance of WAT, is active in mA1 cells [31]. mA2 highly express Etv4, which has been reported to enhance morphological differentiation in preadipocytes [32] (Fig. 2H).

In human adipocytes, the proportion of hA2 was increased samples from type 2 diabetes patients, whereas the proportion of hA1 was reduced (Additional file 1: Fig. S1E). We identified the DEGs of each subpopulation and performed KEGG pathway enrichment. The results showed large functional differences among the three subpopulations. For example, the hA1 subpopulation was characterized by the enrichment of fatty acid degradation and regulation of lipolysis in adipocytes, which can explain the reason for its reduction in diabetes patients (Additional file 1: Fig. S1F).

#### *SAT contains distinct subpopulations of adipocytes*.

**Fig. 3:**
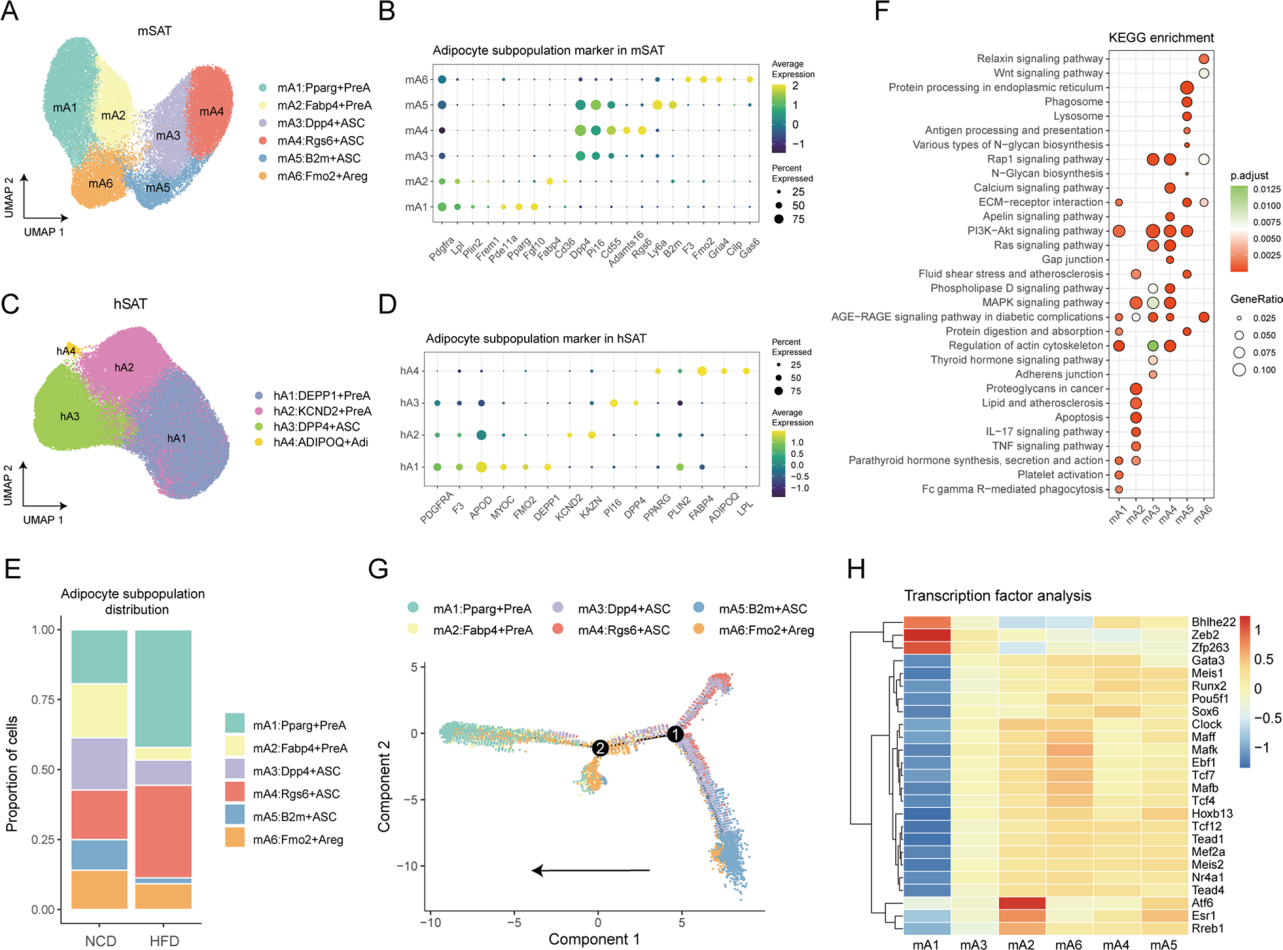
Landscape of the adipocyte population in mouse and human SAT. **A** UMAP visualization of inferred ASPCs from mouse SAT identified six adipocyte subpopulations. **B** Dot plot of marker genes for each cell subpopulations in mouse ASPCs. **C** UMAP visualization of inferred adipocytes from human SAT identified three adipocyte subtypes. **D** Dot plot showing marker genes for each cell subpopulations in human adipocytes. **E** Relative proportions of cell subpopulations in mouse ASPCs from HFD mouse (n = 6) or NCD mouse (n = 22). **F** Dot plot showing the pathway enrichment of each cell subpopulation in mouse ASPCs using KEGG datasets. **G** Pseudo-time trajectory of cell subpopulations in mouse ASPCs. **H** Heatmap showing the highly variable TF activities among the cell subpopulations in mouse ASPCs.

Following a similar procedure, we identified six distinct subpopulations of ASPCs and two subpopulations of mature adipocytes in mSAT (Fig. 3A). Remarkably, all adipocyte subpopulations, with the exception of mA7 and Prune2^+^mature adipocytes in mVAT, were present in mSAT, indicating the robustness of these subtypes. We also identified four subpopulations of human adipocytes, namely, hA1: DEPP1^+^PreA, hA2: KCND2^+^PreA, hA3:DPP4^+^ASC, and hA4: ADIPOQ^+^Adipocyte (Adi), based on their distinct gene expression patterns (Fig. 2C, D). Notably, hA1 and hA2, both expressing APOD and F3, were classified as PreA. While hA1 specifically expressed DEPP1, hA2 expressed KCND2 and KAZN. hA3 was identified as ASC based on the expression of PI16 and DPP4. Of particular interest was the hA4 subpopulation, which exhibited gene expression patterns that are typically associated with mature adipocytes, such as ADIPOQ, LPL, and FABP4 (Fig. 3C, D).

Interestingly, the comparison of the samples with normal and obese states revealed that HFD-induced obesity leads to a significant elevation in the relative number of mA1 and mA4 cells, accompanied by a substantial decrease in the proportion of mA2 and mA5 cells (Fig. 3E). Moreover, we observed a diet-dependent shift in the adipocyte subtypes within mature adipocytes (Additional file 2: Fig. S2A–C).

An important question is whether the functional properties of adipocyte subpopulations are consistent between mSAT and mVAT. To address this question, we performed pathway analysis for markers of each subpopulation (Fig. 3F). mA1 was uniquely enriched in Fc gamma R-mediated phagocytosis, while mA2 was enriched in apoptosis, the TNF signaling pathway and the IL-17 signaling pathway. Among the ASCs, including mA3-5, the mA4 subpopulation was characterized by enrichment in the calcium signaling pathway, gap junction, and apelin signaling pathway. mA5 was uniquely enriched for antigen processing and presentation, suggesting an immune-related feature. mA6, the only Areg subpopulation, was highly enriched in the relaxin signaling pathway and Wnt signaling pathway (Fig. 3F). Next, we performed a cell trajectory analysis of adipocyte subpopulations (Fig. 3G). The results suggested that mA5 is the most stem-like subpopulation in mSAT. mA5 cells can transition through the first branch point to become either mA3 and mA4 cells or and then these cells can through the second branch point to differentiate into mA6 cells. Eventually, they give rise to the mA1 and mA2 PreA subpopulations (Fig. 3G). Finally, we carried out TF analysis of these subpopulations (Fig. 3H). In the results, mA1 showed a relatively particular transcriptional state. Bhlhe22, Zeb2, and Zfp263 were uniquely active in the mA1 subpopulation. In addition, Atf6, Esr1 and Rreb1 were enriched in the mA2 subpopulation (Fig. 3H).

For human adipocytes, the difference in the proportion of subpopulations between samples from obese and lean individuls is minimal. However, obese individuals showed a relatively higher proportion of hA4, and type 2 diabetes patients exhibited a higher proportion of hA4 as well (Additional file 2: Fig. S2D). To gain further insight into the unique functions of each subpopulation, we conducted a thorough analysis of DEGs and subsequently performed KEGG pathway enrichment. Our findings revealed substantial functional divergence among the three subpopulations, as illustrated in Additional file 3: Fig. S3E. The hA4 subtype was uniquely enriched in the PPAR signaling pathway, fatty acid metabolism, AMPK signaling pathway, propanoate metabolism insulin signaling pathway, and regulation of lipolysis in adipocytes (Additional file 3: Fig. S3E).

In summary, we have identified the full range of adipocyte subtypes in mice and presented the molecular profile of each adipocyte subtype in humans. These findings provide valuable insights into the interactions among components in TAME and provide a foundation for further studies on the roles of individual subpopulations in metabolic diseases.

### Communications between adipocyte subpopulations and immune cells

**Fig. 4:**
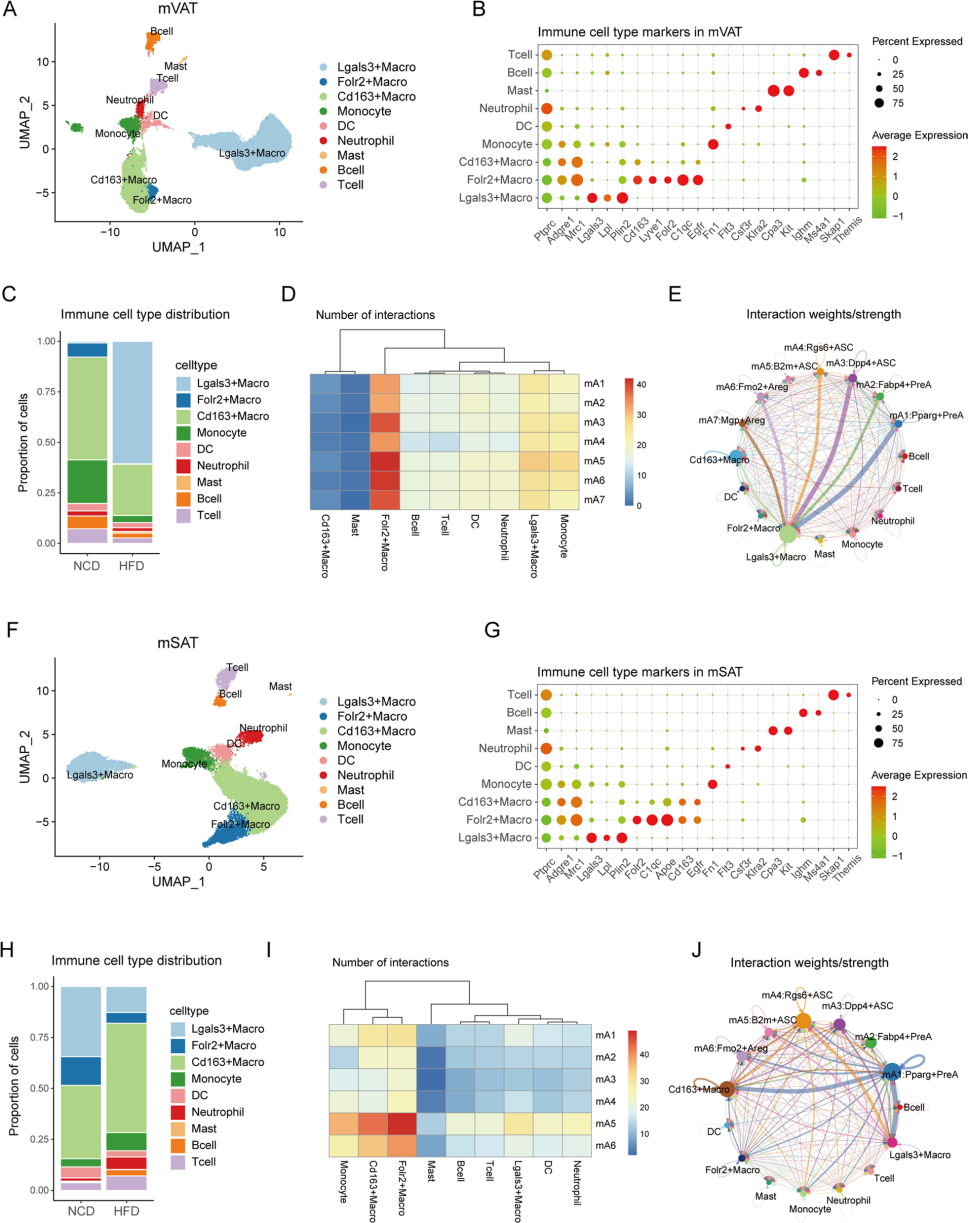
Communications between adipocyte subpopulations and immune cells. **A** UMAP plot showing 9 major immune cell types in mouse VAT. **B** Dot plot of marker genes for each immune cell types in mouse VAT. **C** Relative proportions of immune cell types in mouse VAT from HFD mouse (n = 11) or NCD mouse (n = 8). **D** Heatmap showing the number of the ligand-receptor pairs between each immune cell type and adipocyte subpopulation in mouse VAT. **E** Circos plot showing the weights/strength of interactions between ligands and receptors across cell types in mouse VAT. The size of nodes denotes the weights/strength of pairs involved in each cell type, and the thickness of the line is proportional to the weights/strength of the pairs between two nodes. **F** UMAP plot showing 9 major immune cell types in mouse SAT. **G** Dot plot of marker genes for each immune cell types in mouse VAT. **H** Relative proportions of immune cell types in mouse SAT from HFD mouse (n = 6) or NCD mouse (n = 22). **I** Heatmap showing the number of the ligand-receptor pairs between each immune cell type and adipocyte subpopulation in mouse SAT. **J** Circos plot showing the weights/strength of interactions between ligands and receptors across cell types in mouse SAT. The size of nodes denotes the weights/strength of pairs involved in each cell type, and the thickness of the line is proportional to the weights/strength of the pairs between two nodes

Adipocyte subpopulations communicate with diverse mediators derived from components within the TAME, especially immune cells [33–35]. Thus, we extracted the immune cell populations from mVAT and mSAT to further understand the intricate interplay between adipocytes and immune cells. Reclustering of the immune cell-derived nuclei from mVAT (n = 34,698) resulted in nine subpopulations (Fig. 4A). We performed differential expression analysis and annotated three subpopulations as macrophages (expressing Adgre1 and Mrc1), while the remaining six subpopulations were annotated as monocytes (expressing Fn1), dendritic cells (expressing Flt3), neutrophils (expressing Csf3r and Klra2), mast cells (expressing Cpa3 and Kit), T cells (expressing Themis and Skap1), and B cells (expressing Ms4a1 and Ighm) (Fig. 4A, B). To annotate the macrophage subpopulations, we compared their expression profiles to recent macrophage classification studies. We found that the Lgals3^+^macrophage subpopulation expressed high levels of Lpl and Plin2, consistent with the lipid-associated macrophages (LAMs) that emerge during HFD feeding and play a part in the clearance of dead adipocytes [7, 36]. Folr2^+^macrophages are very similar to tissue-resident macrophages which have been found to reside in the perivascular part of tissues, including WAT [7]. Cd163^+^macrophages expressed a relatively high level of Cd163, suggesting an immunosuppressive feature [37].

Analysis of the frequency of each cell type in mVAT samples from mice fed either a NCD or a HFD revealed an increased proportion of Lgasls3^+^macrophage in samples from HFD-induced obese mice, from almost nonexistent to the most abundant subpopulation. In contrast, the proportion of Folr2^+^macrophages, Cd163^+^macrophages, monocytes, B cells and T cells decreased in the HFD mouse samples (Fig. 4C). Given the significant transition in the distribution and composition of immune cell types within VAT, we next explored to what extent such changes influenced cell communication. Then, we investigated the interactions between ligands and receptors across all immune cells and adipocytes. We found that the number of ligand-receptor pairings between immune cells and adipocyte subpopulations was significantly larger in Folr2^+^macrophages (Fig. 4D), but that the strength of the interactions between Lgals3 + macrophages and adipocytes, especially mA1 and mA3, was stronger (Fig. 4E) [20].

Following similar steps, we reclustered the immune cells derived from the mSAT samples (n = 19,169). We annotated nine immune cell subpopulations that were consistent with those annotated from the mVAT samples (Fig. 4F, G). However, a comparison of the immune cell composition between the normal and HFD-induced obese states revealed a decrease proportion of of Lgals3^+^macrophages and Folr2^+^macrophages (Fig. 4H). And we found that the number of ligand-receptor pairings between immune cells and adipocytes was significantly larger in mA5 and mA6 across all adipocyte subpopulations, especially the mA5: B2m^+^ASC subpopulation (Fig. 4I). Strong signals were detected between mA4/mA5 and Lgals3^+^macrophages, as well as between mA1 and Cd163^+^macrophages (Fig. 4J).

Our findings are consistent with previous studies showing that macrophages are the primary immune cells types in WAT. Additionally, we found that Folr2^+^macrophages act similarly in both visceral and subcutaneous adipose tissue, but the interaction between Folr2^+^ macrophages and adipocytes is not strong and is reduced by obesity. Furthermore, we observed that other communication patterns showed significant depot-specific variance.

### Deconvolution analyses predicted the cancer-associated adipocyte subtypes

We next investigated whether the human TAME retains evidence of adipocyte subtype diversity and which subtype presents tumor-promoting CAA in tumors. To this end, deconvolution analyses were conducted on bulk RNA-seq cohorts of cancer patients from TCGA using the DWLS algorithm [21, 38, 39].

**Fig. 5:**
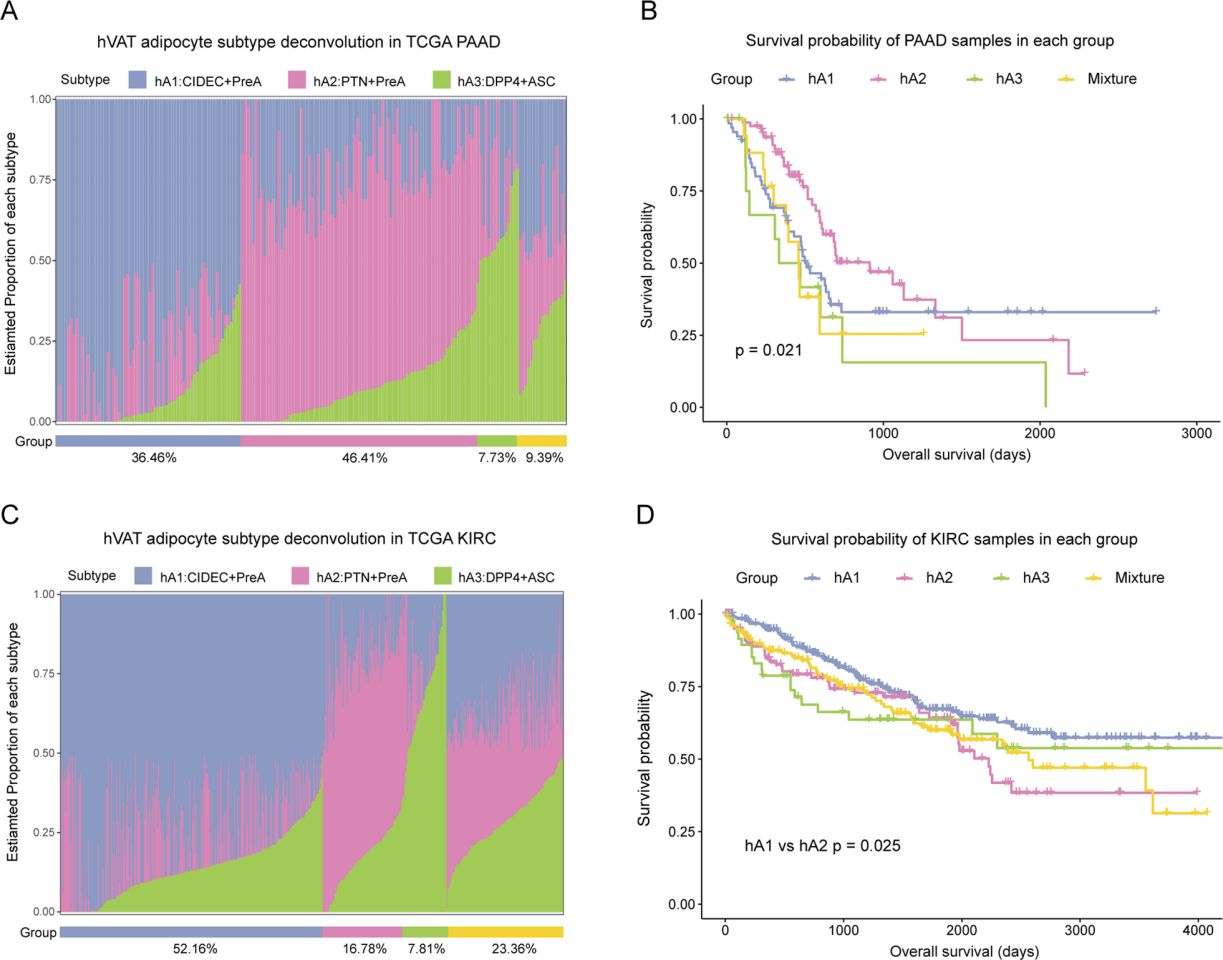
Deconvolution analyses in PAAD and KIRC cohorts. **A** Bar plot showing the estimated proportion of three visceral adipocyte subtypes in each TCGA PAAD sample. **B** Kaplan–Meier survival curve for TCGA pancreatic cancer cohort in four groups. P value was calculated with log‑rank test. Log‑rank p value < 0.05 was considered as statistically significant. **C** Bar plot showing the estimated proportion of the three visceral adipocyte subtypes in each TCGA KIRC sample. **D** Kaplan–Meier survival curve for TCGA KIRC cohort in four groups. P value was calculated with log‑rank test. Log‑rank p value < 0.05 was considered as statistically significant

To assess the hVAT adipocyte subtypes, we estimated individual subtype proportions in pancreatic cancer (PAAD) and kidney clear cell carcinoma (KIRC) patients using our single-cell signatures. Samples from TCGA were divided into four groups based on individual subtype proportions, including hA1, hA2, hA3, and Mixture. The group Mixture comprised samples with multiple subtype components. The PAAD cohort contained 36.46%, 46.42%, 7.73%, and 9.39% of group hA1, hA2, hA3, and Mixture, respectively (Fig. 5A). The group hA3 was associated with a poor OS in pancreatic cancer (Fig. 5B). Additionally, to verify the independent effect of each subtype, we grouped the samples by the estimated scores of each subtype and performed survival analysis. In the PAAD cohort, the hA2 subtype was associated with a promising prognosis, whereas hA3 was associated with a poor prognosis (Additional file 3: Fig. S3A). In the KIRC cohort, hA1 was the dominant subtype, found in 52.16% of the samples, and 16.78%, 7.81%, and 23.36% of samples exhibited hA2, hA3 and Mixture (Fig. 5C). Thereinto, compared with group hA1, the group hA2 was associated with a worse OS (Fig. 5D). In a separate validation of each subtype, the hA1 subtype was associated with a promising prognosis, whereas hA2 and hA3 were associated with a poor prognosis (Additional file 3: Fig. S3B).

**Fig. 6:**
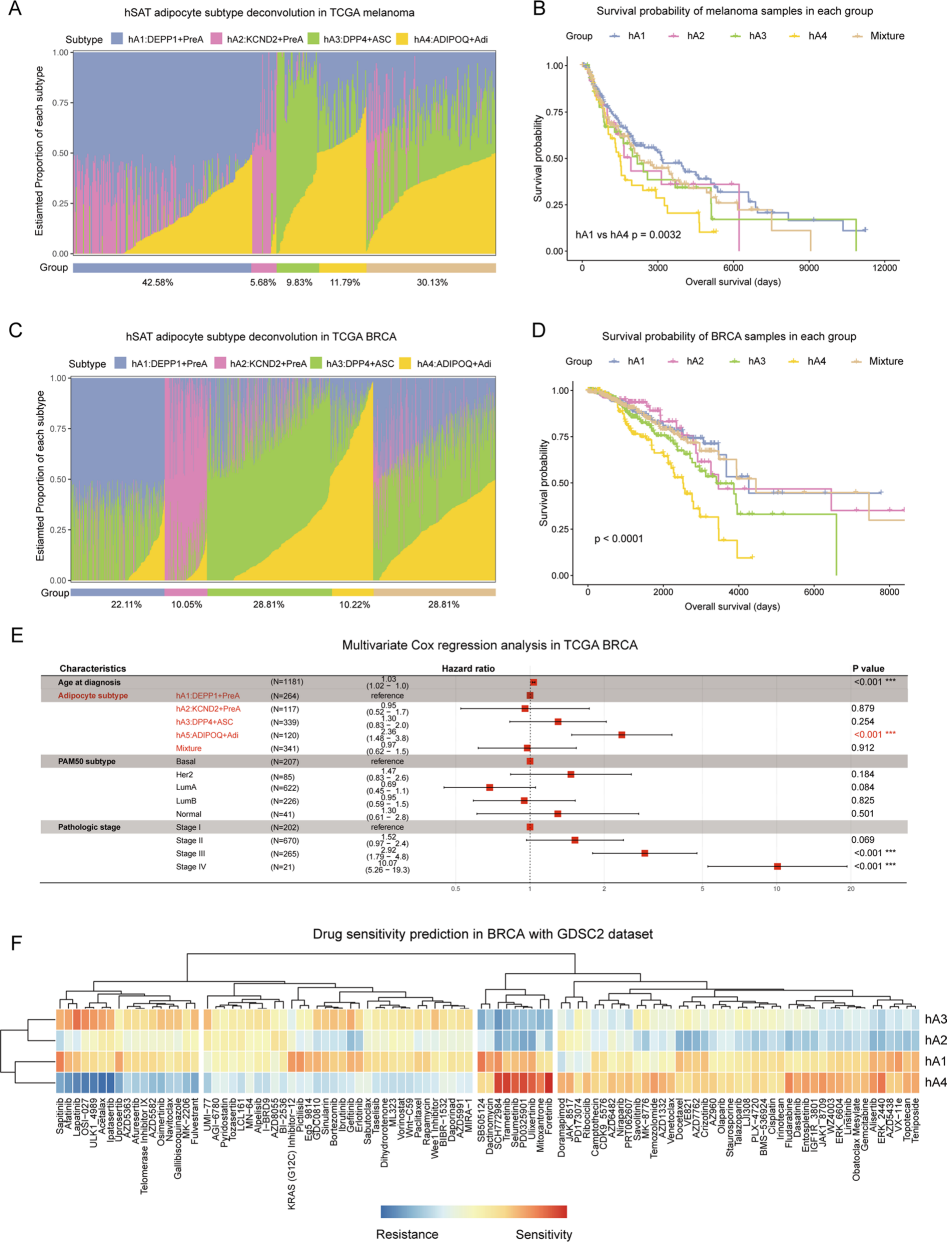
Deconvolution analyses in melanoma and BRCA cohorts. **A** Bar plot showing the estimated proportion of the three subcutaneous adipocyte subtypes in each TCGA melanoma sample. **B** Kaplan–Meier survival curve for TCGA melanoma cohort in five groups. P value was calculated with log‑rank test. Log‑rank p value < 0.05 was considered as statistically significant. **C** Bar plot showing the estimated proportion of the three subcutaneous adipocyte subtypes in each TCGA breast cancer sample. **D** Kaplan–Meier survival curve for TCGA breast cancer cohort in five groups. P value was calculated with log‑rank test. Log‑rank p value < 0.05 was considered as statistically significant. **E** Forest plots for multivariate regression of clinical factors and adipocyte subtypes in TCGA breast cancer datasets. **F** Pearson’s correlation of GDSC2 drug response (measured by IC50) with each of the four subcutaneous adipocyte subtype scores reveals drug resistance (blue) or sensitivity (red)

In the case of hSAT adipocyte subtypes, melanoma and breast cancer (BRCA) cohorts from TCGA were selected as models. Following the same rules, all melanoma cases were categorized into five groups, including hA1, hA2, hA3 hA4, and Mixture (Fig. 6A). Compared to group hA1, group hA4 was associated with worse survival (p = 0.0032) (Fig. 6B). In a separate validation of each subtype, the hA1 subtype was associated with a promising prognosis, whereas the hA3 and hA4 subtype were associated with a poor prognosis (Additional file 4: Fig. S4A). Likewise, all of the breast cancer cases were divided into five groups (Fig. 6C). For survival analysis, group hA4 was associated with the worst survival (p < 0.0001) (Fig. 6D). In addition, to verify the independent effect of each subtype on survival, we performed survival analysis by gene signature score grouping. The hA1 and hA3 subtypes were associated with a promising prognosis, whereas the hA4 subtype was associated with a poor prognosis in breast cancer (Additional file 4: Fig. S4B). The results from Cox regression analyses further confirmed that the hA4 subtype acted as an independent risk factor for breast cancer (Additional file 4: Figs. S4C, 6E).

The hA4 subtype had a significantly worst prognosis in both the melanoma and breast cancer cohorts. Thus, we speculated on the potential drugs targeting the hA4 subtype in breast cancer based on the drug sensitivity data from the GDSC2 [40] and CTRP2 [41] datasets (Fig. 6F). We found that the hA4 subtype was more sensitive to drugs such as SCH772984, trametinib, selumetinib, PD0325901, ulixertinib, mitoxantrone, and foretinib and resistant to drugs such as sapitinib, afatinib, lapatinib, OSI-027, ULK1_4989, acetalaxm and ipatasertib. The hA1 subtype possessed a similar drug sensitivity status to that of the hA4 subtype (Fig. 6F). Using the CTRP2 dataset, several drugs sensitive to the hA4 subtype were also indentified (Additional file 4: Fig. S4D).

In summary, we provided insights into identifying CAA subtypes in four typical cancer types. The DPP4^+^ASC subtype is associated with a poor prognosis in PAAD and KIRC patients, while the ADIPOQ^+^Adi subtype is linked to poor survival in melanoma and BRCA patients. The current findings suggest that trametinib, selumetinib, and ulixertinib may be useful drugs for targeting the hA4 subtype in breast cancer patients.

### AdipoR1 and AdipoR2 mediated breast cancer progression

The above analyses demonstrated that the proportion of the ADIPOQ^+^Adi subtype was elevated in obese and diabetes patients and was associated with poor survival in breast cancer and melanoma patients. To further investigate its regulatory mechanism, we focused on adiponectin (encoded by ADIPOQ) receptors, specifically AdipoR1 and AdipoR2 (encoded by ADIPOR1 and ADIPOR2) [42].

**Fig. 7:**
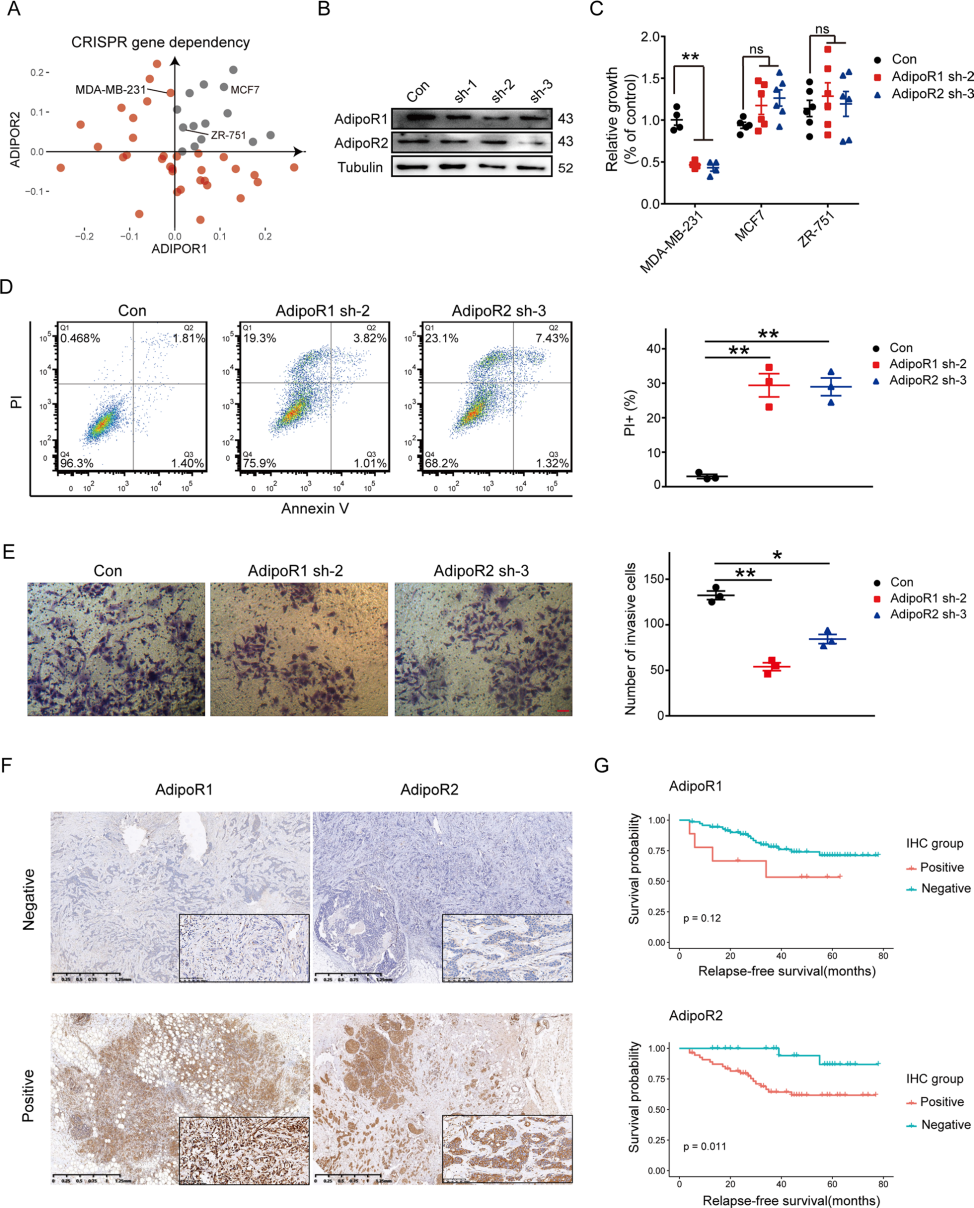
AdipoR1 and AdipoR2 regulates of breast cancer progression. **A** Scatter plot showing the CRISPR gene dependency of ADIPOR1 and ADIPOR2 in breast cancer cell lines. **B** Western blot analysis showing the effect of knockdown by three shRNA sequences in cell line MDA-MB-231. **C** Quantitative analysis showing the proliferation of cell line MDA-MB-231, MCF7 and ZR-751 after knockdown of AdipoR1 and AdipoR2. **D** Flow cytometry results and quantitative analysis showing the cell death level of cell line MDA-MB-231 after knockdown of AdipoR1 and AdipoR2. **E** Transwell assay result and quantitative analysis showing the invasion ability of cell line MDA-MB-231 after knockdown of AdipoR1 and AdipoR2. The number of cells stained by crystal violet under the view of microscope represents the invasiveness of cancer cells. **F** Representative immunohistochemical images of AdipoR1 and AdipoR2 in breast cancer patients. **G** Kaplan–Meier survival curve for breast cancer patients in AdipoR1/2 positive or negative group. P value was calculated with log‑rank test. Log‑rank p value < 0.05 was considered as statistically significant

First, we screened breast cancer cell lines in the Cancer Cell Line Encyclopedia (CCLE) dataset from DepMap [43]. The results showed that the MCF7 and ZR-751 cell lines had high expression of both ADIPOR1 and ADIPOR2 (Additional file 5: Fig. S5A). However, in the gene dependency datasets obtained by the CRISPR screen, the survival/proliferation of the cell line MDA-MB-231, rather than MCF7 and ZR-751, depended on ADIPOR1 (Fig. 7A). Consequently, we selected the MDA-MB-231, MCF7, and ZR-751 cell lines for our in vitro experiments. Our results from western blot analysis showed that sh-2 was more effective in reducing the protein level of AdipoR1, while sh-3 was more effective in reducing the protein level of AdipoR2 (Fig. 7B). To validate the role of AdipoR1/2 in breast cancer cell viability, we conducted MTT assays in the three cell lines. The results indicated that the knockdown of AdipoR1/2 reduced proliferation in the MDA-MB-231cell line, but not in the MCF7 and ZR-751 cell line (Fig. 7C). As a result, we selected the MDA-MB-231 cell line for downstream analyses.

Flow cytometry analysis revealed that the knockdown of AdipoR1/2 promoted cell death in the MDA-MB-231 cell line (Fig. 7D). We then investigated the effect of AdipoR1/2 on the invasiveness of breast cancer cells. Transwell assays results suggested that cell invasiveness was significantly suppressed after the knockdown of AdipoR1 or AdipoR2 (Fig. 7E). Moreover, we validated our conclusion in clinical breast cancer samples. We analysed samples from 113 preoperative breast cancer patients and performed IHC to evaluate the level of AdipoR1 and AdipoR2. The IHC results showed that the levels of AdipoR1 and AdipoR2 varied among patients. We then divided the patients into AdipoR1/2 negative and AdipoR1/2 positive groups based on the estimated IHC score (Fig. 7F). Survival analysis between these two groups showed that AdipoR2 was significantly associated with poor RFS in breast cancer patients (P = 0.011). Moreover, AdipoR1 also showed a negative association with RFS, but the association was not significant (Fig. 7H). We also validated this finding using the METABRIC breast cancer cohort [44]. The expression of both ADIPOR1 and ADIPOR2 was significantly associated with poor OS (Additional file 5: Fig. S5B). Finally, we obtained blood serum samples from 83 preoperative breast cancer patients and found that the level of adiponectin was lower in the overweight group (BMI: 25-29.9) than in the normal group (BMI: 18.5–24.9), but the difference was not significant (p = 0.13) (Additional file 5: : Fig. S5C).

In summary, our findings provided evidence for the positive regulation of AdipoR1 and AdipoR2 in tumor proliferation and invasion in vitro and demonstrated an association between AdipoR1/2 and poor survival in both IHC samples and public datasets. These results suggest that the ADIPOQ^+^CAAs exert a tumor-promoting effect through AdipoR1 and AdipoR2.

## Discussion

This study presents a comprehensive and impartial analysis of the cellular landscape within the adipocyte fraction of mouse and human WAT, thereby enabling us to delineate the in vivo developmental trajectories and characterize the features of adipocyte subpopulations. Based on our adipocyte atlas, we identified cancer-promoting subtypes in TAME using deconvolution approaches. The significance of our study lies in the potential of these CAA subtypes to serve as key prognostic predictors and potential targets for the treatment of adipose-associated cancers. Furthermore, we investigated the tumor-promoting mechanism of ADIPOQ^+^CAAs, which provides a deeper understanding of the role of adipocytes in cancer progression and provides new insights for the development of targeted therapies.

Recent studies have made substantial contributions to our understanding of adipocyte heterogeneity [7, 8]. By integrating multiple datasets, our single-cell atlas reveals a diverse range of adipocyte subpopulations with enhanced robustness. Our analyses identified two PreA, three ASC, two Areg, and three mature adipocyte subpopulations in the mVAT depot, with Rgs6^+^ ASCs being the origin of adipogenesis. Differently, we identified two PreA, three ASC, one Areg, and two mature adipocyte subpopulations in the mSAT depot, with B2m^+^ ASCs serving as the precursor of adipogenesis. What can reach a consensus is that the Pparg^+^PreA subpopulation was the endpoint of adipogenesis in both depots. The origin, composition and distribution of white adipose depots can be substantially different and are influenced by factors such as location, temperature, age, sex, and metabolic disorders such as obesity or diabetes. Several recent studies have focused on the composition and development of the adipocyte population under various conditions [2, 9, 10, 14, 45–47]. For instance, some studies have demonstrated that intrinsic features of ASCs drive the depot-selective ASC hierarchy and *de novo* adipogenesis, potentially explaining why adipogenesis in the cells from HFD-induced obese mice was influenced from the outset in our mVAT atlas. Given the abundance of subpopulations and high-quality sequencing data, we conducted a more in-depth analysis of the mouse datasets. While we found only three or four subpopulations in the human adipocyte population, the mouse WAT atlas will serve as a valuable reference for further enriching the adipocyte map in humans.

Therefore, our objective was to investigate the immune cell population, with a specific focus on macrophages in adipose tissues. Our findings are consistent with previous reports indicating that macrophages are the predominant immune cell type in WAT. In particular, we discovered that Folr2^+^macrophages exhibit similar functions in both visceral and subcutaneous adipose tissue. We observed a substantial number of interactions between Folr2^+^macrophages and adipocytes, although the strength of these interactions varied. Additionally, we identified significant depot-specific differences in other interactions. For example, the interactions between Lgals3^+^macrophages and adipocytes appeared strong in VAT, while further investigation is needed to understand the interactions between Cd163^+^macrophages and adipocytes in SAT. Multiple studies have confirmed the involvement of these cells in the TAME through cellular and animal experiments. It has been reported that depletion of LAMs characterized by the expression of Lgals3 in the TAME synergistically enhances the antitumorigenic effects of anti-PD1 therapy [36]. Furthermore, CD163^+^macrophages were found to be located around adipocytes in breast cancer tissues, and their recruitment and polarization were mediated by the upregulated expression of CCL2 and CCL5 in the TAME [37].

Third, during the identification of CAAs in different cancer types, we observed depot-specific variations in the function of each subtype. For example, Dpp4^+^ASCs, a subtype found in both visceral and subcutaneous adipose tissues, exhibit distinct roles in different cancers. We suggest that DPP4^+^ASCs play a tumor-promoting role in cancers associated with VAT. However, in breast cancer, DPP4^+^ASCs do not serve as an independent risk factor for survival. Additionally, in subcutaneous adipose-associated cancers such as breast cancer and melanoma, the ADIPOQ^+^Adi subtype was associated with poor survival outcomes. Unfortunately, we did not detect the ADIPOQ^+^Adi subtype in the hVAT depot, so we cannot confirm its effect on tumorous associated with VAT. Furthermore, we did not investigate the potential relationship between adipocyte subtypes and other clinical variables, such as lymph node status, metastasis status, pathologic stage, and drug resistance. Previous studies have demonstrated the significant impact of adipocytes on breast cancer progression, including promoting tumor proliferation, migration, and invasion [48]. Adipocytes may also contribute to the growth of estrogen receptor-positive tumors through the upregulation of aromatase expression [49].Additionally, adipocytes may play a role in reducing the efficacy of endocrine and chemotherapy treatments, such as tamoxifen and doxorubicin [50, 51]. Considering the tumor-promoting properties of CAAs, our focus was to explore potential targeted treatment strategies. Specifically, we conducted a drug sensitivity prediction among the four adipocyte subtypes in breast cancer patients and identified potential therapeutic drugs that may target ADIPOQ^+^CAAs. Among these drugs, trametinib, selumetinib, and ulixertinib have been found to inhibit tumor development by targeting the MAPK signaling pathway. However, these drugs are not yet widely used in the clinical management of breast cancer. It is important to note that these drugs target different proteins: trametinib and selumetinib are MEK inhibitors, while ulixertinib is an ERK inhibitor that directly inhibits the MAPK signaling pathway by targeting the ERK protein [52, 53]. We also explored another treatment strategy targeting CAAs at a macroscopic level. Calorie restriction mimetics, such as the IGF1 receptor and isobacachalcone, are drugs that mimic the effects of calorie restriction. These drugs can not only help with weight loss but also enhance immune surveillance in the TAME, making them potential treatments targeting CAAs [54–56].

Ultimately, our aim was to investigate the potential mechanisms underlying the cancer-promoting effects of ADIPOQ^+^CAAs. Adiponectin, which is encoded by ADIPOQ, is secreted by adipocytes and exerts various biological effects. Several studies have suggested that adiponectin has a protective effect against breast cancer [57]. It has been demonstrated that adiponectin induces autophagic cell death in breast cancer through activation of the AMPK-ULK1 axis, which is regulated by STK11/LKB1. Additionally, adiponectin triggers breast cancer cell death through fatty acid metabolic reprogramming [58, 59]. However, not all studies support this notion, as some have found that obesity and decreased adiponectin levels are associated with a higher risk of breast cancer. Adiponectin may act as a growth factor in estrogen receptor-positive breast cancer cells by stimulating cell growth through the MAPK signaling pathway [60]. Nevertheless, in our study, we did not find a significant correlation between serum adiponectin levels and BMI. This finding led us to hypothesize that the high level of adiponectin in TAME, rather than in serum, may contribute to the cancer-promoting effect mediated by ADIPOQ^+^CAAs and AdipoR1/2. Therefore, further research and exploration are necessary to fully understand the role of ADIPOQ and AdipoR1/2 in breast cancer.

Although our study provided valuable insights into the heterogeneity and functional diversity of adipocytes in the TAME, there are some limitations that should be acknowledged. First, we did not directly apply snRNA-seq to CAAs, which may have limited the identification of some CAA subtypes that only exist in the TAME. Second, our study focused solely on the adipocyte population in WAT, and the characteristics and functions of adipocytes in other tissues, such as brown adipose tissue, have yet to be explored. Investigating the heterogeneity of adipocytes in other tissues may provide important insights into their contribution to cancer development. Third, we did not investigate the clinical significance of other adipocyte subtypes, such as the DPP4^+^ASC subtype. Last, while our study identified AdipoR1/2 as potential therapeutic targets in breast cancer, we did not perform in vivo validation of their effects. Further studies utilizing animal models and clinical trials are needed to validate the efficacy of AdipoR1/2 as therapeutic targets and to explore their potential use in breast cancer treatment.

## Conclusions

To sum up, our study provides a perspective that increases our understanding of TAME and enables a deeper exploration of the role of CAAs in cancers. These CAAs may become key prognostic predictors and possible targets for potential treatments in the future. To ensure the comprehensiveness and scientific rigor of our conclusion, we have several ideas for our future work. First, we will continue to investigate the roles and mechanisms of other adipocyte subtypes in breast cancer to understand their potential contributions. Additionally, we will study the roles of these adipocyte subtypes in other types of cancer. Second, we will focus on verifying the feasibility of ADIPOQ^+^CAAs as a potential target for breast cancer treatment, and validate the drugs predicted by our in vivo and in vitro experiments, aiming to provide feasible treatment options for future clinical practice. Finally, we will further explore the signaling pathways by which ADIPOR1/2 promotes breast cancer to better understand the impact of adipocytes on breast cancer.

## Supplementary Information

**Additional file 1: Figure S1.** Supplementary figure for landscape of the adipocyte population in mouse and human VAT. **A** UMAP visualization of inferred mature adipocytes from mouse VAT identified three adipocyte subpopulations. **B** Feature plot of marker genes for each cell subpopulations in mouse mature adipocytes. **C** Relative proportions of cell subpopulations in mouse mature adipocytes from HFD mouse (n = 11) or NCD mouse (n = 8). **D **Pseudo-time trajectory of cell subpopulations in mouse ASPCs marked by diet status. **E** Relative proportions of cell subpopulations in human adipocytes from diabetic (n = 4) or non-diabetic human (n = 15). **F**ot plot showing the pathway enrichment of three cell subpopulations in human adipocytes using KEGG datasets.

**Additional file 2: Figure S2.** Supplementary figure for landscape of the adipocyte population in mouse and human SAT. **A** UMAP visualization of inferred mature adipocytes from mouse SAT identified two adipocyte subpopulations. **B** Feature plot of marker genes for each cell subpopulations in mouse mature adipocytes. **C** Relative proportions of cell subpopulations in mouse mature adipocytes from HFD mouse (n = 6) or NCD mouse (n = 22). **D** elative proportions of cell subpopulations in human adipocytes from obese (n = 23) or lean human (n = 6). **E** Relative proportions of cell subpopulations in human adipocytes from diabetic (n = 5) or non-diabetic human (n = 24). **F** Dot plot showing the pathway enrichment of four cell subpopulations in human adipocytes using KEGG datasets.

**Additional file 3: Figure S3.** Supplementary figure for deconvolution analyses in PAAD and KIRC cohorts. **A** Kaplan–Meier survival curves for TCGA PAAD cohort group by visceral adipocyte subtype score. P value was calculated with log‑rank test. Log‑rank p value < 0.05 was considered as statistically significant. **B** Kaplan–Meier survival curve for TCGA KIRC cohort group by visceral adipocyte subtype score. P value was calculated with log‑rank test. Log‑rank p value < 0.05 was considered as statistically significant.

**Additional file 4: Figure S4.** Supplementary figure for deconvolution analyses in melanoma and BRCA cohorts. **A** Kaplan–Meier survival curves for TCGA melanoma cohort group by subcutaneous adipocyte subtype score. P value was calculated with log‑rank test. Log‑rank p value < 0.05 was considered as statistically significant. **B** Kaplan–Meier survival curve for TCGA BRCA cohort group by subcutaneous adipocyte subtype score. P value was calculated with log‑rank test. Log‑rank p value < 0.05 was considered as statistically significant. **C** Forest plots for univariate regression of adipocyte subtypes in TCGA BRCA cohort. **D** Pearson’s correlation of CTRP2 drug response (measured by IC50) with each of the four subcutaneous adipocyte subtype scores reveals drug resistance (blue) or sensitivity (red).

**Additional file 5: Figure S5.** Supplementary figure for deconvolution analyses in melanoma and BRCA cohorts. **A** Scatter plot showing the expression of ADIPOR1 and ADIPOR2 in breast cancer cell lines. **B** Kaplan–Meier survival curve for METABRIC cohort group by the level of ADIPOR1/2. P value was calculated with log‑rank test. Log‑rank p value < 0.05 was considered as statistically significant. **C** Boxplot showing the blood adiponectin level in patients with different BMI.

## Data Availability

The datasets used and/or analysed during the current study are available from the corresponding author on reasonable request.

## Abbreviations

TAME: Tumor-adipose microenvironment
WAT: White adipose tissue
OS: Overall survival
IHC: Immunohistochemistry
AdipoR: Adiponectin receptor
RFS: Relapse-free survival
SAT: Subcutaneous adipose tissue
VAT: Visceral adipose tissue
ASPC: Adipose stem and progenitor cell
CAA: Cancer-associated adipocyte
scRNA-seq: Single-cell RNA sequencing
snRNA-seq: Single-nucleus RNA sequencing
MNN: Mutual nearest neighbor
UMAP: Uniform manifold approximation and projection
TCGA: The cancer genome atlas
mVAT: Mouse visceral adipose tissue
hVAT: Human visceral adipose tissue
mSAT: Mouse subcutaneous adipose tissue
hSAT: Human subcutaneous adipose tissue
ASC: Adipose stem cell
PreA: Preadipocyte
Areg: Adipogenesis regulator
NCD: Normal control diet
HFD: High-fat diet
TF: Transcription factor
Adi: Adipocyte
PAAD: Pancreatic cancer
KIRC: Kidney clear cell carcinoma
BRCA: Breast cancer
CCLE: Cancer cell line encyclopedia
