# Supplementary figures and images for: Single-cell analysis of white adipose tissue reveals the tumor-promoting adipocyte subtypes
Source: J Transl Med. 2023 Jul 15;21:470. doi: 10.1186/s12967-023-04256-7 (PMC10349475; doi:10.1186/s12967-023-04256-7)

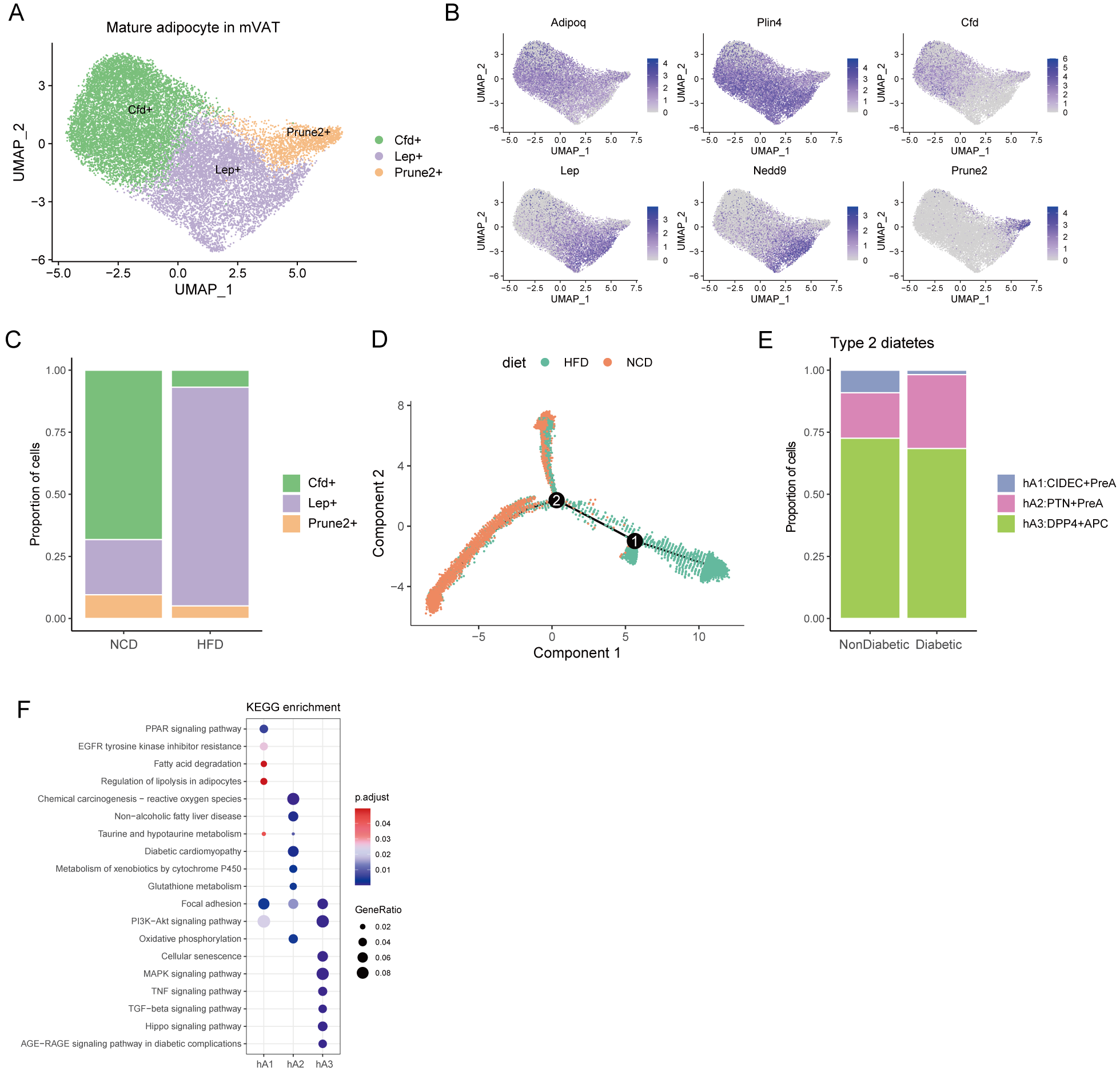

Supplement: Supplementary file 1 — Additional file 1: Figure S1. Supplementary figure for landscape of the adipocyte population in mouse and human VAT. A UMAP visualization of inferred mature adipocytes from mouse VAT identified three adipocyte subpopulations. B Feature plot of marker genes for each cell subpopulations in mouse mature adipocytes. C Relative proportions of cell subpopulations in mouse mature adipocytes from HFD mouse (n = 11) or NCD mouse (n = 8). D Pseudo-time trajectory of cell subpopulations in mouse ASPCs marked by diet status. E Relative proportions of cell subpopulations in human adipocytes from diabetic (n = 4) or non-diabetic human (n = 15). Fot plot showing the pathway enrichment of three cell subpopulations in human adipocytes using KEGG datasets. [file 12967_2023_4256_MOESM1_ESM.tif]

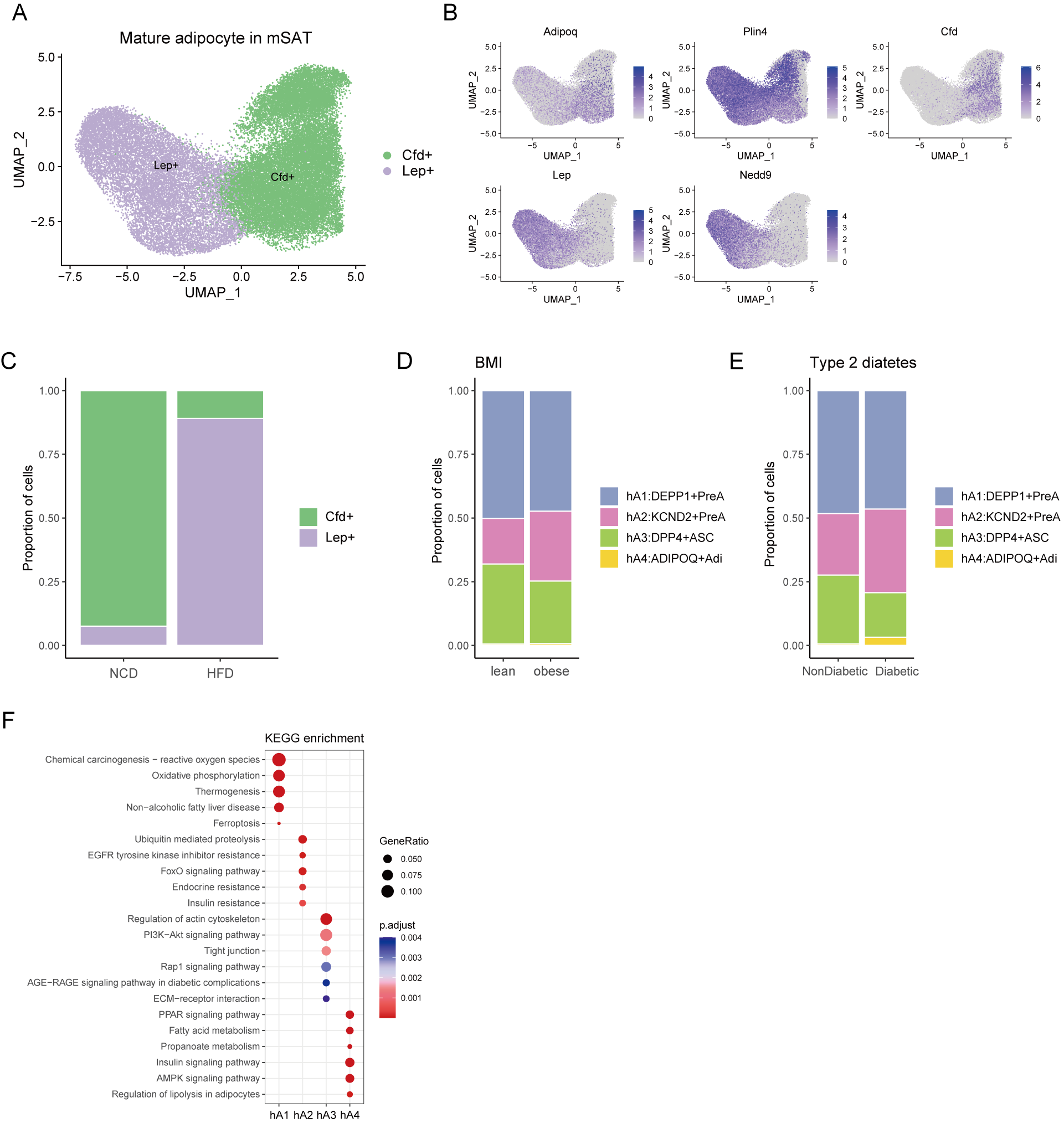

Supplement: Supplementary file 2 — Additional file 2: Figure S2. Supplementary figure for landscape of the adipocyte population in mouse and human SAT. A UMAP visualization of inferred mature adipocytes from mouse SAT identified two adipocyte subpopulations. B Feature plot of marker genes for each cell subpopulations in mouse mature adipocytes. C Relative proportions of cell subpopulations in mouse mature adipocytes from HFD mouse (n = 6) or NCD mouse (n = 22). D elative proportions of cell subpopulations in human adipocytes from obese (n = 23) or lean human (n = 6). E Relative proportions of cell subpopulations in human adipocytes from diabetic (n = 5) or non-diabetic human (n = 24). F Dot plot showing the pathway enrichment of four cell subpopulations in human adipocytes using KEGG datasets. [file 12967_2023_4256_MOESM2_ESM.tif]

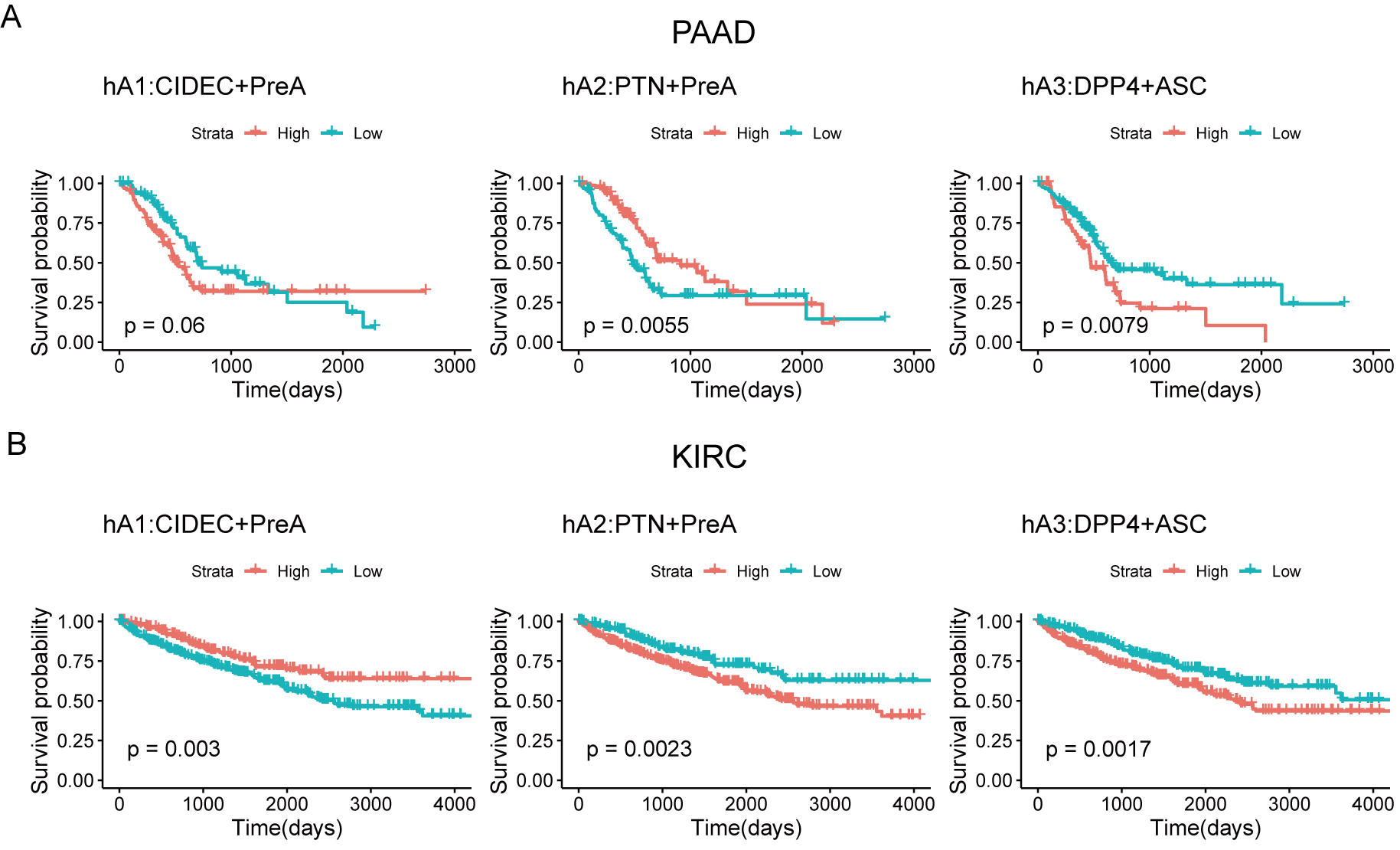

Supplement: Supplementary file 3 — Additional file 3: Figure S3. Supplementary figure for deconvolution analyses in PAAD and KIRC cohorts. A Kaplan–Meier survival curves for TCGA PAAD cohort group by visceral adipocyte subtype score. P value was calculated with log‑rank test. Log‑rank p value < 0.05 was considered as statistically significant. B Kaplan–Meier survival curve for TCGA KIRC cohort group by visceral adipocyte subtype score. P value was calculated with log‑rank test. Log‑rank p value < 0.05 was considered as statistically significant. [file 12967_2023_4256_MOESM3_ESM.tif]

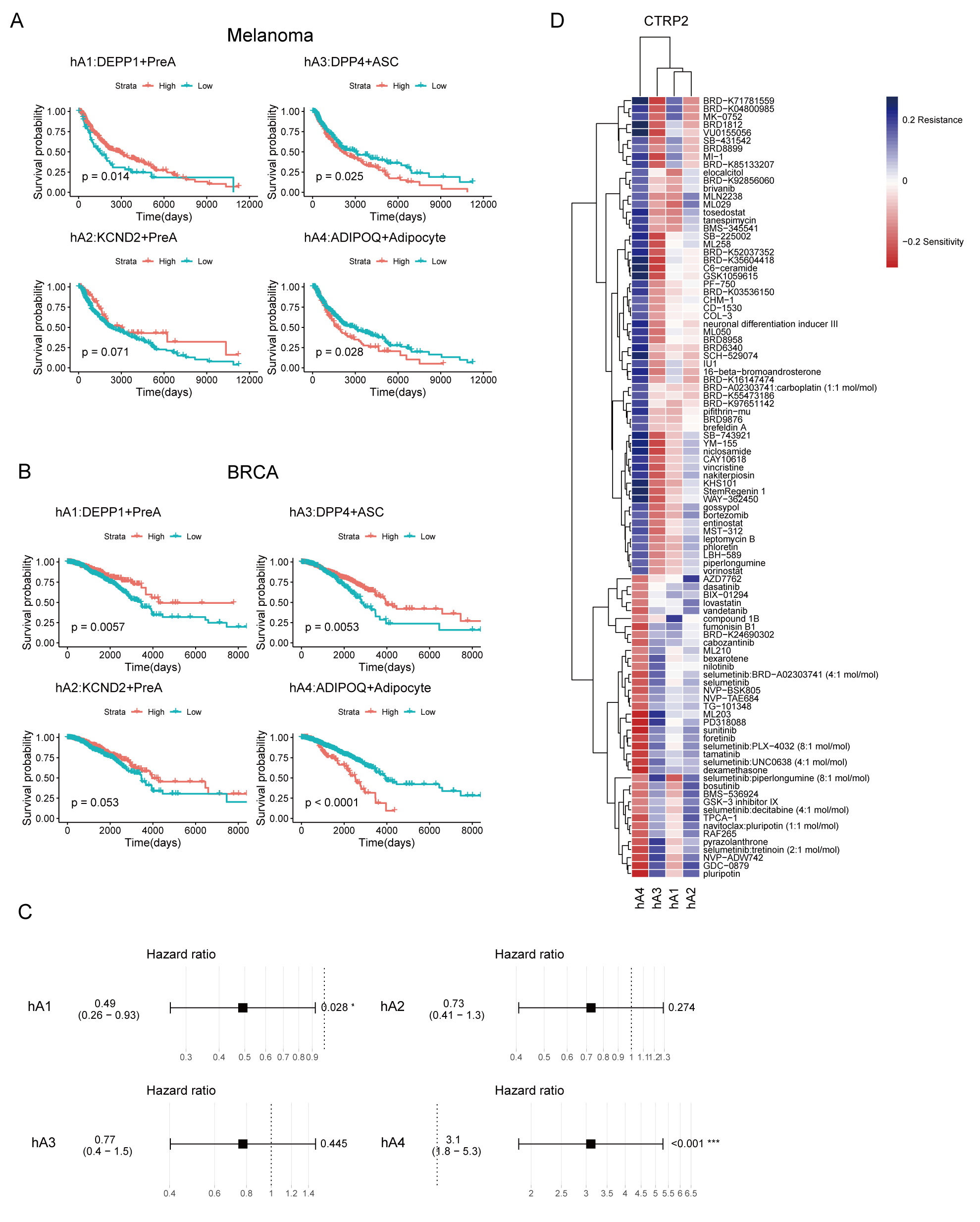

Supplement: Supplementary file 4 — Additional file 4: Figure S4. Supplementary figure for deconvolution analyses in melanoma and BRCA cohorts. A Kaplan–Meier survival curves for TCGA melanoma cohort group by subcutaneous adipocyte subtype score. P value was calculated with log‑rank test. Log‑rank p value < 0.05 was considered as statistically significant. B Kaplan–Meier survival curve for TCGA BRCA cohort group by subcutaneous adipocyte subtype score. P value was calculated with log‑rank test. Log‑rank p value < 0.05 was considered as statistically significant. C Forest plots for univariate regression of adipocyte subtypes in TCGA BRCA cohort. D Pearson’s correlation of CTRP2 drug response (measured by IC50) with each of the four subcutaneous adipocyte subtype scores reveals drug resistance (blue) or sensitivity (red). [file 12967_2023_4256_MOESM4_ESM.tif]

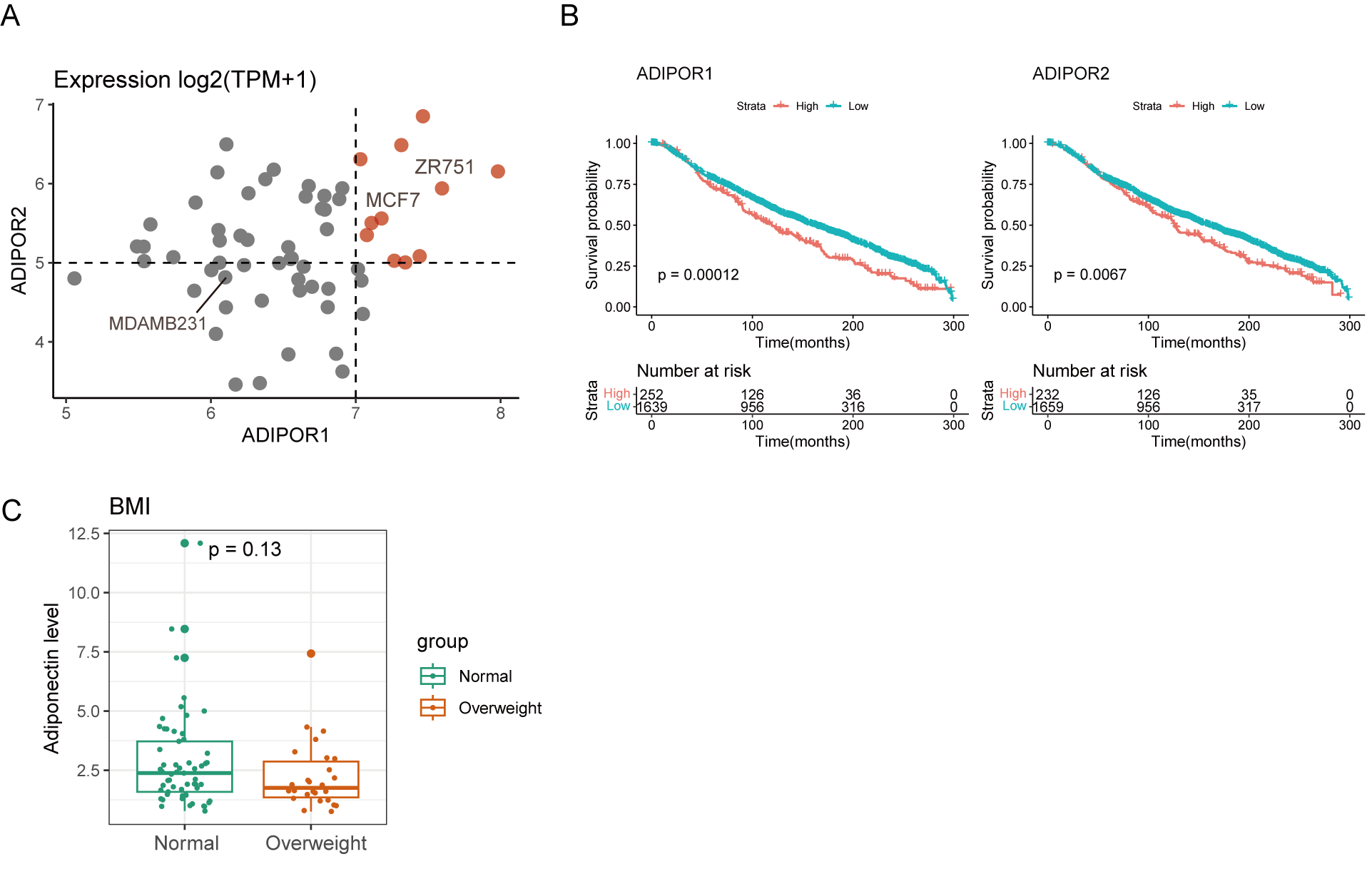

Supplement: Supplementary file 5 — Additional file 5: Figure S5. Supplementary figure for deconvolution analyses in melanoma and BRCA cohorts. A Scatter plot showing the expression of ADIPOR1 and ADIPOR2 in breast cancer cell lines. B Kaplan–Meier survival curve for METABRIC cohort group by the level of ADIPOR1/2. P value was calculated with log‑rank test. Log‑rank p value < 0.05 was considered as statistically significant. C Boxplot showing the blood adiponectin level in patients with different BMI. [file 12967_2023_4256_MOESM5_ESM.tif]
